# Stability of dissolved and soluble Fe(II) in shelf sediment pore waters and release to an oxic water column

**DOI:** 10.1007/s10533-017-0309-x

**Published:** 2017-02-27

**Authors:** J. K. Klar, W. B. Homoky, P. J. Statham, A. J. Birchill, E. L. Harris, E. M. S. Woodward, B. Silburn, M. J. Cooper, R. H. James, D. P. Connelly, F. Chever, A. Lichtschlag, C. Graves

**Affiliations:** 1 Ocean and Earth Science, National Oceanography Centre, University of Southampton, Southampton, SO14 3ZH UK; 2 Present Address: LEGOS, Université de Toulouse, CNES, CNRS, IRD, UPS, 14 Avenue Edouard Belin, 31400 Toulouse, France; 3 Department of Earth Sciences, University of Oxford, South Parks Road, Oxford, OX1 3AN UK; 4 School of Geography, Earth and Environmental Science, University of Plymouth, Drake Circus, Plymouth, PL4 8AA UK; 5 Plymouth Marine Laboratory, Prospect Place, The Hoe, Plymouth, PL1 3DH UK; 6 Centre for Environment, Fisheries and Aquaculture Science, Pakefield Road, Lowestoft, NR33 0HT UK; 7 Marine Geosciences, National Oceanography Centre, European Way, Southampton, SO14 3ZH UK

**Keywords:** Benthic iron flux, Shelf sediment, Oxic shelf, Porewaters, Iron isotopes, Redox speciation, Seasonality, Ligands

## Abstract

Shelf sediments underlying temperate and oxic waters of the Celtic Sea (NW European Shelf) were found to have shallow oxygen penetrations depths from late spring to late summer (2.2–5.8 mm below seafloor) with the shallowest during/after the spring-bloom (mid-April to mid-May) when the organic carbon content was highest. Sediment porewater dissolved iron (dFe, <0.15 µm) mainly (>85%) consisted of Fe(II) and gradually increased from 0.4 to 15 μM at the sediment surface to ~100–170 µM at about 6 cm depth. During the late spring this Fe(II) was found to be mainly present as soluble Fe(II) (>85% sFe, <0.02 µm). Sub-surface dFe(II) maxima were enriched in light isotopes (δ^56^Fe −2.0 to −1.5‰), which is attributed to dissimilatory iron reduction (DIR) during the bacterial decomposition of organic matter. As porewater Fe(II) was oxidised to insoluble Fe(III) in the surface sediment layer, residual Fe(II) was further enriched in light isotopes (down to −3.0‰). Ferrozine-reactive Fe(II) was found in surface porewaters and in overlying core top waters, and was highest in the late spring period. Shipboard experiments showed that depletion of bottom water oxygen in late spring can lead to a substantial release of Fe(II). Reoxygenation of bottom water caused this Fe(II) to be rapidly lost from solution, but residual dFe(II) and dFe(III) remained (12 and 33 nM) after >7 h. Iron(II) oxidation experiments in core top and bottom waters also showed removal from solution but at rates up to 5-times slower than predicted from theoretical reaction kinetics. These data imply the presence of ligands capable of complexing Fe(II) and supressing oxidation. The lower oxidation rate allows more time for the diffusion of Fe(II) from the sediments into the overlying water column. Modelling indicates significant diffusive fluxes of Fe(II) (on the order of 23–31 µmol m^−2^ day^−1^) are possible during late spring when oxygen penetration depths are shallow, and pore water Fe(II) concentrations are highest. In the water column this stabilised Fe(II) will gradually be oxidised and become part of the dFe(III) pool. Thus oxic continental shelves can supply dFe to the water column, which is enhanced during a small period of the year after phytoplankton bloom events when organic matter is transferred to the seafloor. This input is based on conservative assumptions for solute exchange (diffusion-reaction), whereas (bio)physical advection and resuspension events are likely to accelerate these solute exchanges in shelf-seas.

**Electronic supplementary material:**

The online version of this article (doi:10.1007/s10533-017-0309-x) contains supplementary material, which is available to authorized users.

## Introduction

Whilst iron (Fe) is a major constituent of the solid Earth, dissolved Fe (dFe) concentrations in oxic waters are very low (typically <1 nM) because of the limited solubility of oxidised forms of the metal. However, this element and its redox cycling have major impacts on the cycling of other elements (e.g., P) in terrestrial waters and during diagenesis in sediments (Raiswell and Canfield 2012). Iron is also an essential micro-nutrient for marine primary producers and limits their growth in ~25% of the open ocean (Boyd and Ellwood 2010), due to its low availability. As marine primary productivity plays a significant role in CO_2_ uptake from the atmosphere, Fe is argued to be important in the regulation of the global climate (Sigman and Boyle 2000). It is therefore important to understand how Fe is supplied to the ocean from different sources, and how it is cycled and removed. Typically, Fe concentrations are highest close to sources (atmospheric inputs, hydrothermal vents, rivers and the seafloor). For shallow shelf and slope waters, rivers are anticipated to be only a small source because of extensive removal of Fe in low salinity waters during estuarine mixing (Sholkovitz et al. 1978). Atmospheric inputs are most significant where dust inputs are large, and are directly delivered to the photic zone (de Jong et al. 2007). However, sediments underlying shelf and slope waters are argued to be a major source of Fe that can ultimately be transferred to the ocean (Johnson et al. 1999; Elrod et al. 2004; Severmann et al. 2010; Homoky et al. 2012; Conway and John 2014; Dale et al. 2015).

Within marine sediments, dissimilatory iron reduction (DIR) by bacteria can produce large inventories of soluble Fe(II) in ferruginous zones of interstitial porewaters during the decomposition of organic matter (Burdige 2006). These Fe(II) maxima carry diagnostically light isotopic signals, typically −2.0 to −1.0‰ (Severmann et al. 2006, 2010; Homoky et al. 2009). It is widely accepted that towards the oxic sediment-water boundary Fe(II) is oxidised to Fe(III), and lost to insoluble Fe(III) oxides. This oxidative trap prevents transfer of most of the sediment sourced dFe to oxic seawater. This process is associated with the removal of heavy Fe isotopes leading to further enrichment of light Fe isotopes in the porewater (Severmann et al. 2006; Homoky et al. 2009). Within more oxidizing sediments, the isotopic signature of dFe is close to crustal compositions, e.g., 0.0–0.2‰ (Homoky et al. 2009, 2013), and the dFe fraction consists mainly of Fe colloids (0.02–0.2 μm), that are argued to originate from “non-reductive” dissolution processes (Radic et al. 2011; Homoky et al. 2011, 2013). The Fe isotopic composition in the water column may serve as a tool to trace benthic Fe fluxes which originate from different dissolution processes and are transported to the ocean interior (Conway and John 2014).

The processes involved in the supply of Fe from sediments to shelf seas, and its fate in the water column, are not yet fully understood. Benthic chamber measurements have shown that the largest fluxes of dFe are released from continental margin sediments with high inventories of organic carbon and reactive iron minerals underlying oxygen-depleted waters (Severmann et al. 2010; Dale et al. 2015). However, few studies have examined the Fe cycle in oxic shelf seas, even though they represent a large fraction of the ocean’s continental boundaries (Homoky et al. 2016). There are many reports of oxic shelf waters with higher dissolved Fe (dFe) than the open ocean (Charette et al. 2007; Cullen et al. 2009; de Jong et al. 2012; Marsay et al. 2014) and hence they may also be a significant source to the ocean in the global Fe cycle.

The mechanisms involved in the supply of Fe from sediments to the overlying water include diffusion of Fe from porewaters, sediment resuspension by bottom boundary currents, or tidal currents and bio-turbation and bio-irrigation. Whilst a geochemical overview exists for the cycling of Fe in marine sediments (e.g., Burdige 2006) details of processes that can lead to transfer of dFe to oxic shelf seas are lacking. From a seasonal perspective in temperate systems, large amounts of organic matter of bloom origin are deposited onto the seafloor during spring. The organic matter gradually decomposes, leading to the release of remineralised nutrients and Fe into porewaters and across the sediment-water interface. However, to date, there are no data that document the seasonal changes in porewater Fe in such systems, or the controls on dFe exchange between sediments and the water column. It is crucial to obtain estimates of Fe fluxes from different types of shelf environments to better constrain global supply rates in biogeochemical models that feed into global climate models (Homoky et al. 2016).

This study is part of an extensive research programme in the Celtic Sea (Thompson et al. submitted). The Celtic Sea is a temperate, shallow (<200 m) and oxic shelf sea, which undergoes deep mixing during winter and becomes more stratified in summer (Williams et al. 2013; Thompson et al. submitted). The shelf system has a broad connection to the adjacent North Atlantic Ocean, and provides an ideal setting to study processes controlling the sedimentary dissolution and release of Fe to the overlying water column. Here we present a seasonal study of Fe cycling within cohesive sediments of the Celtic Sea shelf, examining the form and fate of Fe in porewaters, and mechanisms whereby this Fe may be released to overlying waters.

## Materials and methods

### Sampling of sediments, porewaters and seawater

All laboratory apparatus, filters and sample bottles used for low concentration Fe determinations were cleaned using a rigorous acid washing regime, including different dilutions of HCl (up to 2 M) and ultrapure purified water (Milli-Q, resistivity of 18.2 MΩ cm).

**Fig. 1:**
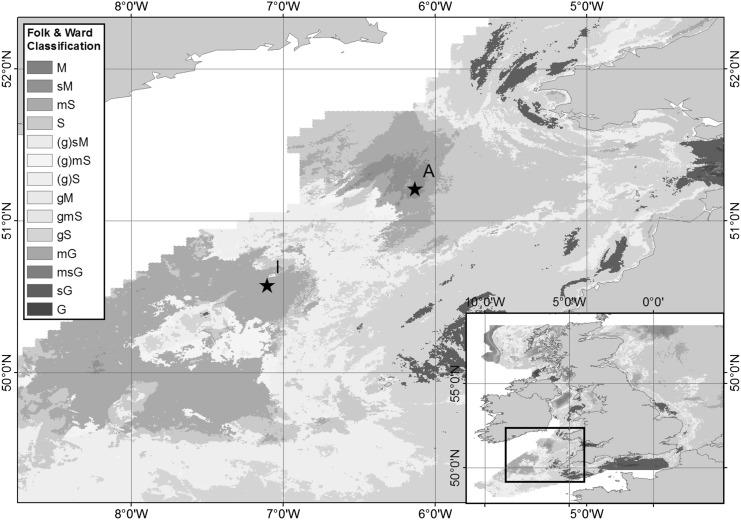
Location of study sites *A* and *I* (*black stars*) on a map of surface sediment types for the UK shelf (*inset*) and Celtic Sea areas using simplified Folk textural classifications from mud (M) through sand (S) to gravel (G), based on surface sediment maps of the British Geological Survey (Folk 1954; Stephens 2015; Stephens and Diesing 2015). Site A is located in an area of sandy mud (sM) and Site I is located in an area of muddy sand (mS). Adapted from Thompson et al. (submitted)

Sediments were collected from cohesive Site A (sandy mud ~51°12.6754′N, 6°8.0277′W) and Site I (muddy sand ~50°34.5557N, 7°6.3161W) within the Celtic Sea (Fig. 1). Sediments from these sites distinguish themselves in mean grain size and porosity (for more details, see Thompson et al. submitted). This study mainly focuses on Site A sediments, while a couple of experiments were run with sediments from Site I. Sediments were collected using pre-drilled clear polycarbonate core tubes (60 cm length, 10 cm diameter) mounted on a Bowers and Connelly Mega Corer, which minimises sediment disturbance during collection. The Celtic Sea was revisited twice during 2015, on the RRS *Discovery* cruises DY030 (4th to 25th May, 2015, late spring conditions) and DY034 (6th August to 2nd September, 2015, late summer conditions). The spring bloom started in early April, had fully developed bloom conditions on the 19th of April and lasted until mid-May. Therefore, DY030 captures part of the end of the bloom and post-bloom conditions and DY034 occurred 2–3 months after the bloom. On each visit sediment cores with clear overlying water were selected and taken into a temperature-controlled laboratory (set to bottom water temperature) on the vessel. The overlying water was siphoned through Teflon and Tygon tubes into a low-density polyethelyene (LDPE) bottle, and immediately filtered through 0.2 μm filters (Cyclopore, Whatman) in a Nalgene filtration unit. Each filtrate was sub-sampled for dFe, dFe isotopes and nutrients into LDPE bottles. Oxygen depth profiles were then measured by microsensors (see below) and any residual core top water was siphoned to waste.

With core top water removed, porewaters were extracted from sediments cores at 1 to 2 cm depth intervals using Rhizon samplers (Seeberg-Elverfeldt et al. 2005). The MOM-type Rhizon samplers (Rhizosphere Research Products B.V., 50 mm long, 2.5 mm diameter, 0.15 μm pore size) were inserted at right angles through PVC tape that covered the pre-drilled holes in the core tube walls, and attached via luer-lock fittings to nitrile-free syringes (20 ml, BD Discardit). At each depth, suction was applied and then, after discarding the first 0.5 ml to waste, a porewater sample was collected. This approach allows for the fast extraction and handling of porewater samples (Homoky et al. 2013), and effectively isolates redox sensitive trace metals from oxidation artefacts. Subsample aliquots were immediately fixed with the synthetic Fe(II) ligand ferrozine (3-(2-pyridyl)-5,6-diphenyl-1,2,4-triazine) for ship-board Fe(II) and total Fe analyses (details below). Porewater subsamples for dFe isotopes and dMn were stored in clean LDPE bottles and acidified to pH 1.8 with distilled concentrated HCl. Subsamples for nutrients were taken for ship-board analysis. During cruise DY030, the soluble size fraction (<0.02 μm, sFe) of porewaters was also collected through a nitrogen gas-purged Anotop25 syringe filter (Whatman), of which subsamples were immediately fixed with ferrozine for ship-board Fe(II) and total Fe analyses, and subsamples stored for sMn.

After porewater extraction, the residual sediment of one core from each season was sliced using a polycarbonate sheet at 0.5, 1 and 2 cm depth-intervals, and stored at −20 °C in zip-lock bags prior to further analyses.

Water column samples were collected using a titanium rosette fitted with 24 × 10 L Ocean Test Equipment (OTE) water samplers adapted for trace metal work and coupled to a conductivity-temperature-depth (CTD) system (Seabird 911+), as well as oxygen, transmissiometer and fluorometer sensors (rosette hereafter called Ti-CTD). All sampling procedures followed GEOTRACES protocols as reported in the *Sampling and Sample* -*handling Protocols for GEOTRACES Cruises* (http://www.geotraces.org/).

### Analytical methods

Analytical methods are described in detail in the Supplementary Information. Oxygen profiles in sediment cores and O_2_ monitoring in Fe(II) oxidation experiments were measured with Unisense O_2_ micro sensors. The limit of detection (LOD, three times the standard deviation of the blank) was ≤0.3 μM.

The concentrations of Fe species—Fe(II) and Fe(II) plus Fe(III) (hereafter total Fe)—were determined in the dissolved and soluble size fractions of porewater samples using the Fe(II)-complexing ferrozine ligand (Sigma-Aldrich) (Stookey 1970; Viollier et al. 2000). Concentrations >1 μM Fe(II) were measured on a spectrophotometer (ATI Unicam 8625) and concentrations <1 μM Fe(II) were measured on a 250 cm 3000 Series Liquid Waveguide Capillary Cell (LWCC, World Precision Instruments) (Waterbury et al. 1997). On the spectrophotometer, the LOD was 0.3 μM Fe(II) and the blank was 0.25 μM Fe(II). The typical relative standard deviation (obtained by measuring replicates) was 2% for >10 μM and up to 5% below 4 μM. For the LWCC, the LOD was 0.7 nM, the blank was 6 ± 4 nM and the typical relative standard deviation was <5%.

The concentration of dFe(II) in the water column was determined by flow injection chemiluminescence using luminol (Bowie et al. 2002; Ussher et al. 2007). The LOD of this method (defined as three times the standard deviation of the blank) was 15 pM Fe(II) and the blank was 25 pM Fe(II). The average relative standard deviation, obtained from triplicate analyses, for all samples above the LOD was 6.7%.

A ^57^Fe/^58^Fe double spike technique was used to determine the isotopic composition of Fe in porewaters, seawater samples and core-top waters (Lacan et al. 2010; Conway et al. 2013). Samples were analysed on a Neptune *Plus* (Thermo Scientific) with an external reproducibility of ±0.04‰ (2 SD).

Porewater Mn concentrations in soluble and dissolved size fractions were determined in diluted samples on a quadrupole ICP-MS (X-Series, Thermo Scientific). The blank was <0.8 nM, the LOD was 0.2 nM and the typical relative standard deviation was 2%.

In order to examine phase associations of Fe and Mn in solid sediment phases, two leaching schemes were applied to one core per season: (i) an ascorbic acid leach (Raiswell et al. 2010) was applied, to extract the easily reducible oxide phases, such as amorphous ferrihydrite, but not the more crystalline oxide phases, and (ii) an acetic acid-hydroxylamine-HCl (H-HCl) leach (Berger et al. 2008), that extracts other amorphous oxide phases as well as ferrihydrite. Fe and Mn in the leach solutions were determined using an inductively coupled plasma optical emission spectrometer (ICP-OES, iCAP6000 Series, Thermo Scientific). For Fe, the blank was <0.06 μg/g, the LOD was <4 μg/g and the typical standard deviation was ~20 μg/g. For Mn, the blank was <0.02 μg/g, the LOD was <0.6 μg/g and the typical standard deviation was 4 μg/g.

Nutrient concentrations in water column samples, in sediment porewaters and experiments, were all analysed on board using a Bran and Luebbe segmented flow colorimetric auto-analyser (Woodward and Rees 2001). The typical relative standard deviation was 2–3%; and the LODs were 0.02, 0.01, and 0.05 µmol l^−1^ for “nitrate plus nitrite”, nitrite, and ammonia, respectively. Sulphide was measured using the colorimetric technique of Cline (1969), and the detection limit was 1 µM.

Particulate organic carbon (POC) and nitrogen (PON) were determined using a Carlo-Erba CHNOS analyser (Nieuwenhuize et al. 1994). Precision was 6.6% RSD at 1.5% POC and 2.4% RSD at 0.13% PON. Estimated detection limit for C was 3.6 µg and all sample carbon contents were well above this value.

### Shipboard experiments

In order to study conditions that are potentially important for the transfer of dFe across the sediment surface, a series of shipboard micro-cosm experiments were carried out on the late spring and late summer cruises. Experiments were performed in the ship’s constant temperature laboratory set to ambient bottom water conditions, and used acid washed (>1 M HCl) equipment and analytical procedures adapted for low concentration Fe analyses described previously.

The first experiment was designed to assess the qualitative impact of periodic water column deoxygenation on the diffusive release of dFe and dFe(II) from sediment cores to overlying water, and further to see how rapidly Fe would be lost upon re-oxygenation of the overlying water. The experiment was run on both, late spring and late summer sediment cores and water samples from Site A (Fig. 1). One sediment core and its overlying bottom water was sealed from the ambient atmosphere and the depletion of oxygen was induced—due to microbial respiration within the experiment—for about a week in the dark. Then, the sealed core was opened and aerated, while kept in the dark. During aeration the oxygen concentration in overlying water was monitored continuously, and sub-samples of seawater were used to assess dFe(II) and dFe at several time intervals during this re-oxidation processes over a period of ~2 days.

The second set of experiments was designed to measure the rates of dFe(II) oxidation in selected bottom waters in the presence and absence of sediments. The experiments were carried out in late summer only, on bottom waters collected with the titanium rosette ~10 m above the seafloor, and on bottom water overlying sediment cores (two replicates) collected at Site I (muddy sand) (Fig. 1). An Fe(II) standard (diluted from ammonium Fe(II) sulphate hexahydrate, Sigma Aldrich, purum p.a. grade) was added to these water samples for a target concentration of 200 nM Fe(II). The Fe(II) spiked waters were kept in the dark to ensure no photo-reduction of Fe(III) occurred, and the waters were sub-sampled (typically 15 times) over a period of ~4 h to monitor the rates at which dFe(II) decreased over time.

The third experiment (carried out during late summer only) investigated any diffusive flux of dFe(II) and dFe across the sediment water interface, by periodically subsampling core top water overlying sediments, which were collected from Site I (Fig. 1). For further details, see Supplementary Information.

## Results

**Fig. 2:**
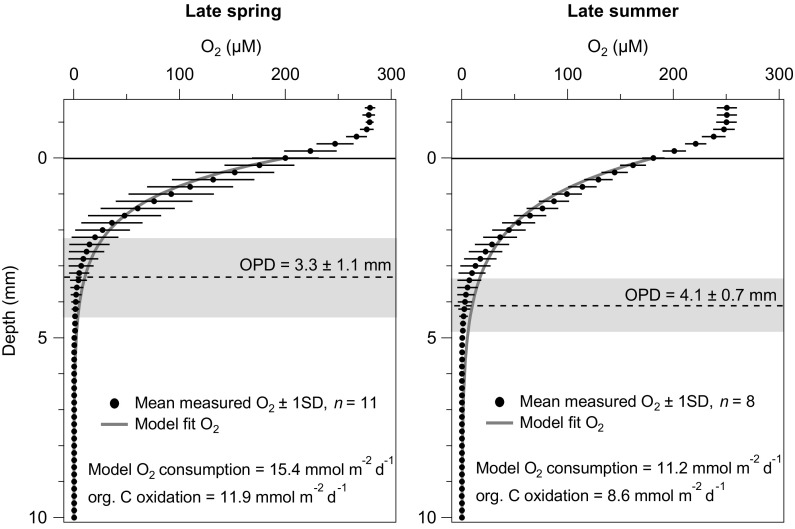
Oxygen depth-profiles across the sediment-water interface at Site A during late spring and late summer. *Symbols* show seasonally averaged O_2_ measurements, and *error bars* (2 SD, *n* = 8–11) reflect natural variations down core. Analytical precision of individual measurements is within the width of the symbols. *Dashed lines* reflect mean O_2_ penetration depths (OPDs) and *grey bars* their variability in each season (2 SD, *n* = 8–11). Diffusive boundary layers overlying the sediment water interface are observed to be 0.6 mm. Modelled fits for oxygen depth-profiles are shown by the *solid orange line*; modelled seasonally averaged O_2_ consumption and proportional rates of seasonally averaged organic C oxidation rates are presented (see text for more details)

The shallowest and most variable oxygen penetration depths (OPDs) were observed in late-spring at our study site (Site A), coincident with the end of the spring bloom. OPDs ranged from 2.2 to 5.8 mm during the late spring (average 3.3 ± 1.1 mm, *n* = 11) and from 3.4 to 5.6 mm during late summer (average 4.1 ± 0.7 mm, *n* = 8; Fig. 2). Based on replicate determinations of OPD, a t-test (0.07) strengthens our view that OPDs were significantly (*p* < 0.1) shoaled in late spring compared to late summer. We used a one-dimensional steady-state oxygen diffusion-consumption model to approximate the rates of organic C oxidation by fitting calculated outputs to the observed mean oxygen depth profile from each season. The reduction of squared residuals between modelled and observed values was used to optimize the model fit. The approach follows (Berner 1980), in which a single pool of reactive organic C is attributed to oxygen consumption and the influences of bioturbation, seasonal sediment accumulation and porosity structure are ignored, as described elsewhere (e.g., Papadimitriou et al. 2004; Homoky et al. 2013). Oxygen consumption rates are found to be highest in the late-spring, corresponding to a proportionally higher rate of organic carbon oxidation in the sediments (11.9 mmol m^−2^ day^−1^) compared to the late summer season (8.6 mmol m^−2^ day^−1^; Fig. 2).

**Fig. 3:**
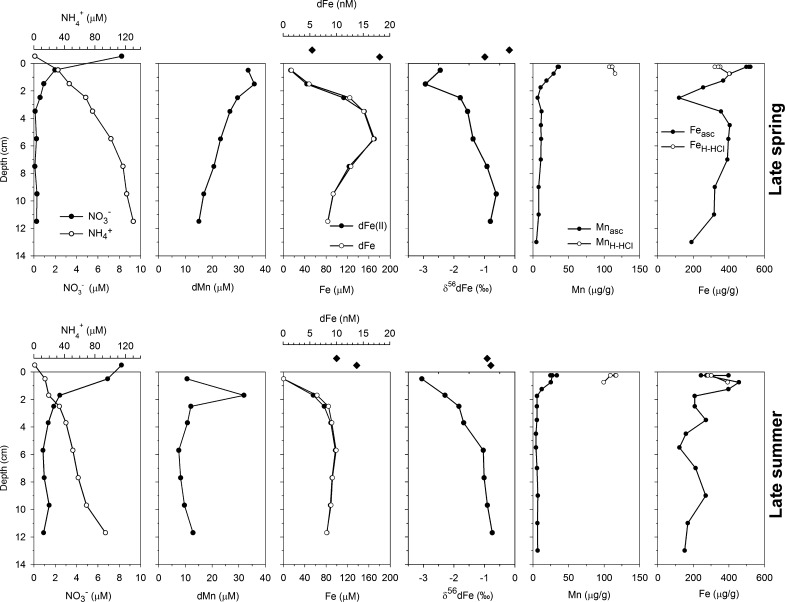
Distribution of NO_3_^−^, NH_4_^+^, dMn, dFe, dFe(II), and δ^56^Fe in porewaters and bottom waters and distribution of Fe and Mn in easily reducible oxide phases in sediments. Parameters are displayed for one selected core from each season. Concentrations of NO_3_^−^ and NH_4_^+^ in core top water (bottom water sampled from sediment cores) are displayed above the sediment-water interface; core top water N0_3_^−^ and NH_4_^+^ concentrations in late spring refer to averaged values from Cores H and C (see Supplementary Information), as these data are not available for this core. *Diamond*-*shaped* symbols above the sediment-water interface show dFe concentrations and isotopic compositions from core top water and bottom seawater (sampled ~10 m above the seafloor); note the change in axis for Fe concentrations in pore water (μM) and bottom water (nM). All *error bars* (SD) are smaller than the symbols

Porewater nitrate maxima were similar in late spring and late summer seasons, between 1 and 7 μM in the surface 0–1 cm (Fig. 3). Porewater ammonia increases with depth in both seasons consistent with nitrate conversion to ammonia. Nitrate concentrations were higher in bottom waters (~8 μM; Fig. 3), whereas ammonia concentrations were lower.

**Fig. 4:**
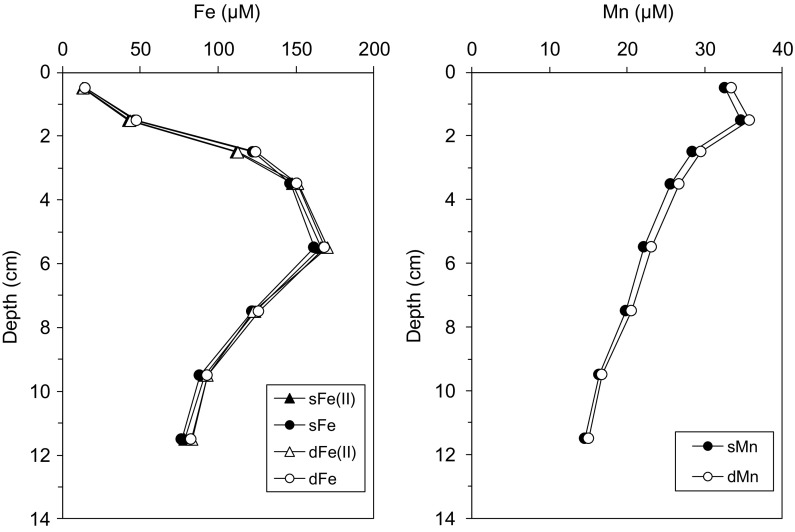
Distributions of soluble and dissolved Fe and Mn concentrations in porewaters during late spring. *Error bars* (1 SD) are within marker shapes

Porewater Mn concentrations had similar trends during both seasons, with shallow sub-surface maxima at 1–2 cm (Fig. 3). Porewater Mn in the soluble size fraction (sMn) was also measured during the late spring season and had similar concentrations to dissolved Mn (dMn; Fig. 4), indicating that dMn was entirely in reduced and soluble forms, consistent with the reductive dissolution of Mn(IV) following nitrate consumption.

**Fig. 5:**
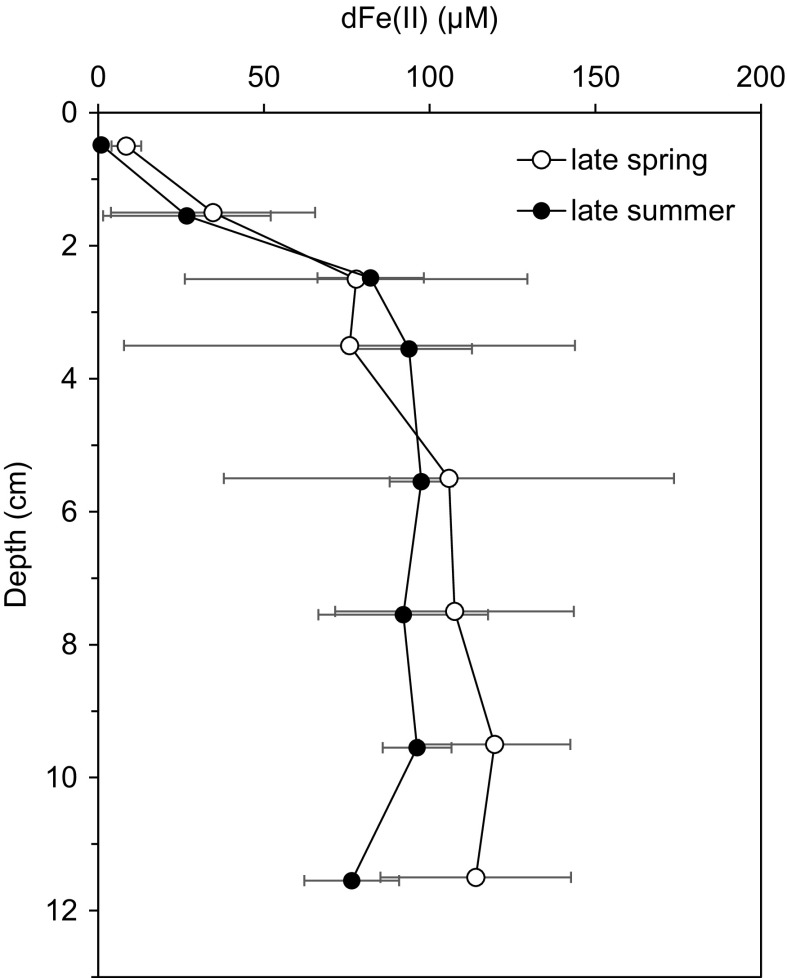
Average porewater dFe(II) concentrations during late spring and late summer. Averages are from three measurements of replicate sediment cores and *error bars* are the standard deviation of these three measurements, dominantly reflecting natural heterogeneity within the sediment. Between seasons porewater dFe(II) concentrations are indistinguishable from natural heterogeneity below 1 cm depth. However, during late spring, above 1 cm depth dFe(II) concentrations are significantly higher than during the late summer

**Fig. 6:**
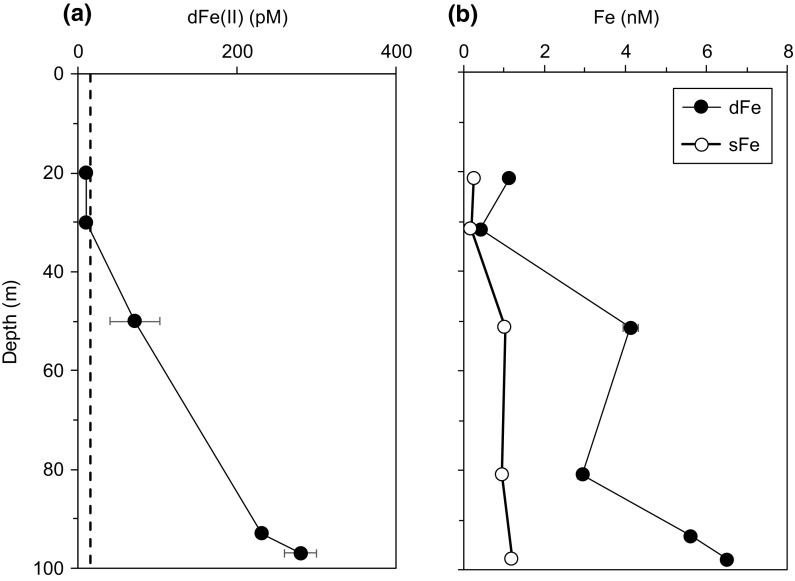
Distributions of **a** dFe(II) and **b** dFe and sFe (Birchill, personal communication) in the water column at Site A during June 2015 (cruise DY033). The *vertical dotted line* indicates LOD for Fe(II) measurements and error bars indicate the standard deviation of each measurement

Porewaters had similar depth-profiles for Fe concentration across all cores in both late spring and late summer (Fig. 3; additional profiles are displayed in the Supplementary Information). Dissolved Fe was low at the surface (0.3–13 μM, 0–1 cm), and steadily increased towards large sub-surface maxima (100–170 μM, 5–8 cm), before decreasing slightly further down-core (~80 μM, 12 cm depth). Generally, porewater total dFe equals dFe(II) within sampling and analytical uncertainties. In addition, soluble Fe species (sFe), which were measured only during the late spring conditions, correspond with dFe concentrations within analytical uncertainty (Fig. 4). However, surface and sub-surface dFe(II) concentrations were greatest in the late spring. Sub-surface dFe(II) maxima were 150 ± 20 μM (*n* = 3) in the late spring compared to 110 ± 10 μM (*n* = 3) in late summer conditions. Uppermost surface porewater dFe(II) concentrations were significantly elevated in late spring conditions (5–13 μM) compared to late summer conditions (0.3–1.2 μM; Fig. 5). Concentrations decreased by 2-5 orders of magnitude in core top waters (14–21 nM; Fig. 3; Table S4), relative to dFe in surface porewater, and decreased further into bottom waters approximately 10 m above the seafloor (5.4–10 nM; Figs. 3, 6; Tables S4, S5).

The isotopic composition of dFe (δ^56^dFe) in porewater depth-profiles was similar in both seasons, ranging from −0.6‰ at 10–12 cm, to −3.0‰ in uppermost sediments—comparatively lighter than core top water and bottom waters, where δ^56^dFe values ranged from −1.0 to −0.1‰ (Fig. 3).

Porewater sulphide (H_2_S) was below the limit of detection (<1 μM) in our samples. By proxy, the abundance of dFe(II) in our deepest porewaters is also consistent with the absence of any substantial sulphate reduction and production of sulphide, and even trace amounts of H_2_S below our detection limit would likely have already been titrated as Fe-sulphide by the excess of dFe(II).

Easily leached forms of Fe and Mn were consistently higher in surface sediments above dissolved sub-surface maxima during both seasons (Fig. 3), and indicate a significant fraction of porewater Fe and Mn entrapment must be forming these authigenic solid-phases (Fig. 3). Ascorbic acid released Fe (Fe_asc_) was highest in surface sediments (up to 520 µg/g) and decreased down-core to ~150 µg/g. Trends for ascorbic released Mn (Mn_asc_) were similar (surface up to ~35 μg/g decreasing to ~5 μg/g at depth). The H-HCl leach performed in the surface 1 cm of sediment resulted in a similar Fe release to that of the ascorbic acid leach. However, the H-HCl leach removed significantly more Mn (up to 120 μg/g) compared to the ascorbic acid leach.

Water column dFe(II) at our study site was also found to increase significantly towards the seafloor from <15 pM at <30 m (~75 m above the sea floor, asf) to 280 pM at 97 m (10 m asf) when measured between our sampling seasons, in July 2015 (Fig. 6). Furthermore, Fe(II) represents ~4% of the total dFe pool in these bottom waters (Birchill, personal communication), and is suggestive of a benthic source of dFe(II) and dFe to the water-column.

**Fig. 7:**
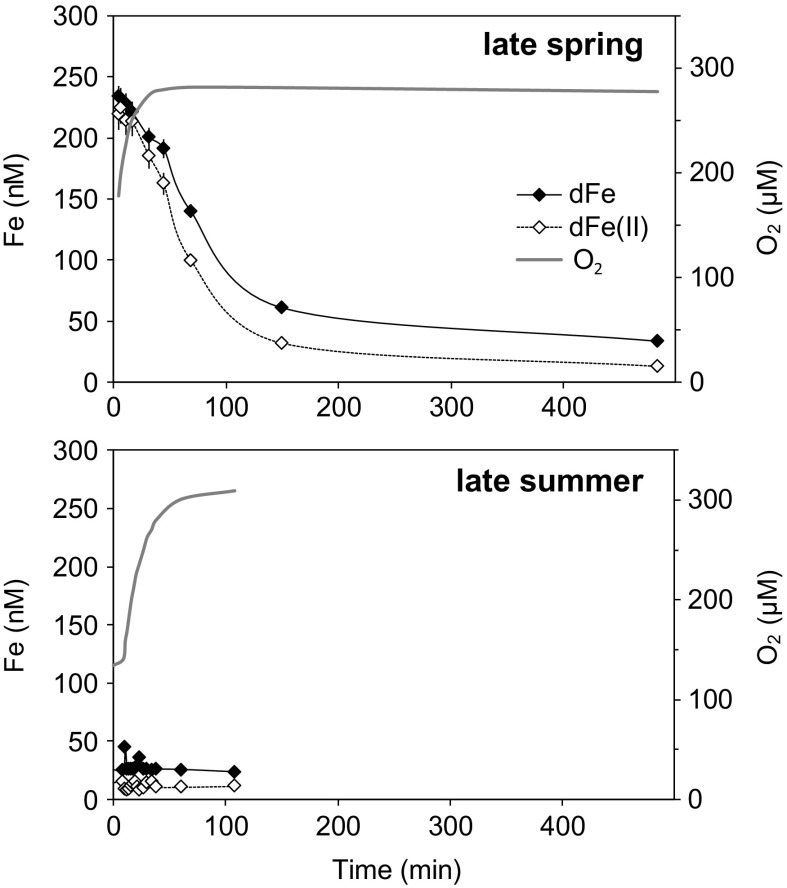
Concentrations of dFe and dFe(II) in core top water over time during its reoxygenation after forcing decreased oxygen conditions in the late spring and the late summer. *Error bars* are standard deviations of single measurements

The air-tight incubation of water overlying sediment cores resulted in substantially elevated dFe(II) and dFe concentrations in the late spring (up to 225 and 232 nM, respectively), whilst in late summer much lower concentrations of dFe(II) and dFe were detected (on average 12 and 28 nM, respectively; Fig. 7). During the subsequent ventilation of these incubated core top waters in the late spring, dFe(II) concentrations decreased 10-fold after 150 min, but after nearly 500 min dFe(II) and dFe persisted at elevated concentrations similar to the late summer experiment (12 and 33 nM, respectively). Ventilation of core top water in the late summer produced no impact on dFe(II) or dFe concentrations in core top waters (Fig. 7). The pH was monitored during the summer experiment and did not vary significantly (7.73 ± 0.05, 1 SD, *n* = 2).

**Fig. 8:**
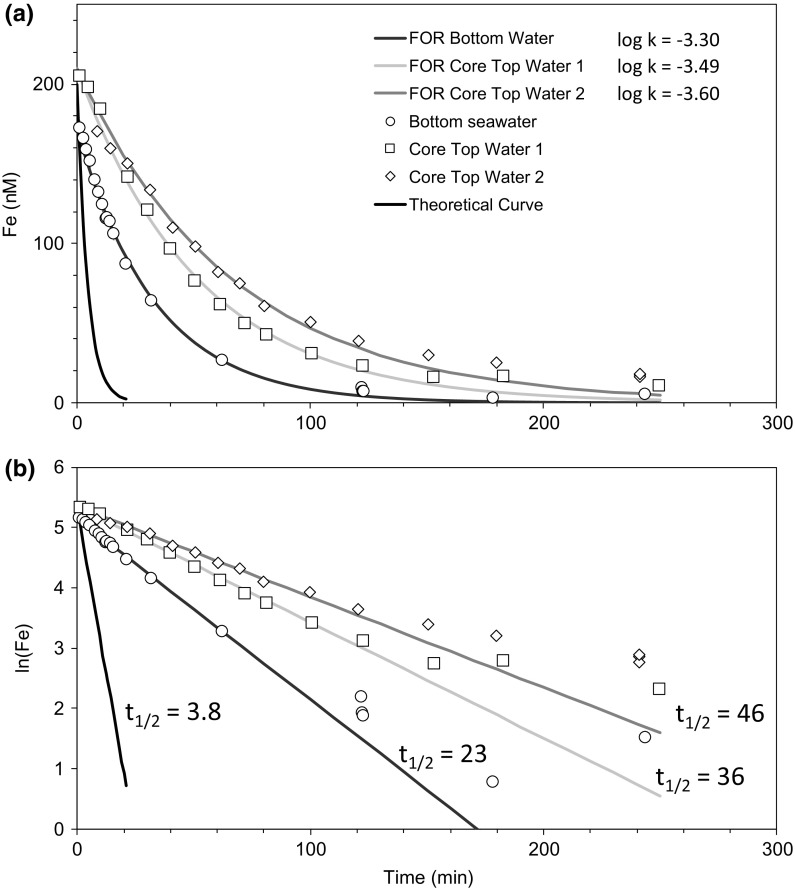
Iron(II) oxidation kinetics, plotted against **(a)** Fe (nM) and **(b)** ln(Fe), in isolated bottom seawater and in bottom water overlying sediment cores (Core top water, replicates 1 and 2) in late summer conditions. Theoretical predictions (Millero et al. 1987) are displayed for comparison. The first-order rate constant, *k*, has been adjusted to find the best fit of data to a first-order rate (FOR) equation (plot **a**), [Fe]_t_ = [Fe]_0_ × e^−*k*t^, from which the half-lives, t_1/2_ (min), are displayed in plot (**b**)

Further, Fe(II) oxidation experiments were designed to compare Fe(II) oxidation kinetics from core top waters in contact with sediments, bottom water isolated from sediments, and theoretical rates (Fig. 8). All waters exhibited Fe(II) oxidation rates slower than theoretical predictions based on empirical constraints for seawater (Millero et al. 1987), with core top waters presenting the slowest rates of Fe(II) loss. Measurements of Fe(II) in diffusion experiments in the late summer showed no significant increase of dFe(II) or dFe over a period of 3 days (dFe(II) <2 nM; dFe <4 nM, Figure S2), which confirms the absence of significant inputs of Fe due to diffusion or handling in our shipboard experiments. The pH was monitored throughout the experiments and remained at 8.1 ± 0.3 (1 SD, *n* = 5).

## Discussion

### Seasonal redox cycling of dFe and sFe in Celtic Sea shelf sediments

Oxygen penetration depths were most varied but, on average, shallowest in late spring (2.2–5.8 mm, Fig. 2), which is consistent with wider spatio-temporal assessments of OPD in the Celtic Sea (Hicks et al. submitted). Most likely, this reflects the enhanced supply of organic carbon to the sediment and metabolic consumption of O_2_, combined with enhanced macro benthic faunal activity at our study site. Higher rates of oxygen consumption and organic carbon oxidation are supported by modelled diffusion-consumption of O_2_ (Fig. 2). In addition, this hypothesis is supported by peak chlorophyll abundance, observed via MODIS satellites two weeks prior to our sediment sampling in late spring (Thompson et al. submitted), and particulate organic carbon concentrations in the surface layers (0–10 cm) that are highest in late spring (1.25%), and decrease by late summer (1.14%) as bloom-derived carbon is decomposed (Figure S1).

Beneath the sediment surface, dissolved macronutrients (NO_3_^−^, NH_4_^+^) and metals (Fe, Mn) in porewaters follow their anticipated biogeochemical depth-distributions during early diagenesis (Froelich et al. 1979; Burdige 2006). Following the consumption of O_2_, NO_3_^−^ is reduced and transformed to NH_4_^+^. The benthic N-cycle is in reality complex but, briefly, the remineralisation of organic matter leads to the release of nitrogen in the form of NH_4_^+^, which is immediately oxidised to NO_3_^−^ in the presence of oxygen, and then removed via denitrification and anammox in anoxic sediments below (e.g., Devol et al. 2015).  Nitrate reduction is followed by the reduction of solid Mn and Fe oxides down-core and the release of their soluble reduced forms to porewaters (Fig. 2).

To date, there is little detailed knowledge of the redox state and size distributions of Fe in shelf sediment porewaters. Here we demonstrate that the proportion of porewater dFe as dFe(II) at our study site is >85% in the upper 3 cm and ~100% in the ferruginous zone below (Fig. 2). Thus, Fe(II) is supplied to porewater in the sub-surface (from the dFe(II) maxima, between 3 and 8 cm depth) and is prone to oxidative-removal towards the sediment surface, and most likely to sulphide-mineral trapping at deeper depths, resulting in the curvature typical of porewater Fe(II) profiles (Froelich et al. 1979; Severmann et al. 2006). Porewater profiles of Fe and Mn and their seasonal variations are similar to those in depositional areas of the southern and eastern North Sea (Slomp et al. 1997). Porewater dFe maxima are isotopically light (δ^56^Fe −1.7 to −0.9‰), consistent with supply of dFe(II) from the dissimilatory reduction of Fe(III) oxide by bacteria (Severmann et al. 2006; Homoky et al. 2009; Henkel et al. 2016). In addition, our observations of dFe, δ^56^dFe, dMn, nitrate and POC content in the Celtic Sea are similar to observed ranges in sediments from low-oxygen California-Oregon shelves (Severmann et al. 2006; Homoky et al. 2009, 2012) where benthic fluxes of isotopically light dFe to the ocean are observed (Severmann et al. 2010).

For the first time we have combined physicochemical Fe observations in porewater with speciation measurements. We show that porewater dFe(II) is almost entirely (>85%) in the soluble (<0.02 µm) size range, and colloidal forms of Fe are largely absent under these conditions (Fig. 4). Similarly, porewater dMn(II) is also found in the truly soluble phase (100% of dMn is <0.02 μm). The sFe forms may be simple ionic species, nano-particulate forms or may contain small ferrozine-reactive Fe(II) organic complexes. This contrasts with findings from deep sea sediments in the Crozet region, where on average 80% of Fe and 61% of Mn was in the colloidal size fraction (0.02–0.2 μm) (Homoky et al. 2009, 2011). Porewater dFe mainly being in the reduced form in the shelf sediments studied here also means that there is no evidence for any significant concentrations of Fe(III) organic complexes as reported for anoxic porewaters in estuarine systems (Jones et al. 2011; Beckler et al. 2015).

Dissolved Fe generated in sediments and supplied to porewater will diffuse along its concentration gradient towards regions of reactive consumption or transport loss. Iron(II) oxidation in porewaters may be coupled to O_2_ or NO_3_^−^ reduction (Laufer et al. 2016). There are good empirical basis’ to understand Fe(II) oxidation kinetics attributed to O_2_ (Millero et al. 1987) and in the presence of NO_3_^−^ (e.g., González et al. 2010), previously explored in a study of benthic Fe flux (Homoky et al. 2012). However, the impact of enzymatic Fe(II) oxidation via NO_3_^−^ reduction is still unclear. Near-surface gradients in dFe and dMn clearly indicate diffusion towards reaction in the surface oxic-layer, and potentially to the overlying water-column. A concomitant increase in sediment-leachable Fe and Mn is seen towards the sediment-water interface (Fig. 3), and accounts for an important fraction of dissolved Fe and Mn removal in surface sediments. A similar inverse correlation between porewater dFe(II) and hydroxylamine-HCl leachable Fe has been observed in surface sediment from the North Sea, where the leachable Fe pool was also suggested to originate from sub-surface DIR (Henkel et al. 2016). The ascorbic acid leach extracts easily reducible ferrihydrite, which is the first amorphous Fe oxyhydroxide phase precipitated due to Fe(II) oxidation (Raiswell et al. 2010). The hydroxylamine-HCl leach extracts ferrihydrite as well as other reactive Fe phases that have been argued to be bioavailable (Berger et al. 2008). Manganese seems to be trapped preferentially by materials released by the reducing hydroxylamine-HCl leach.

No sulphide was detected at any depths in our porewaters, and we observe negligible down-core enrichment of heavy Fe isotopes in porewater that would be indicative of removal to sulphides (Severmann et al. 2006). However, the gradual decrease in dFe(II) below its maximum (Fig. 3) most likely reflects downward diffusion as dFe(II) is converted to FeS in an underlying sulphate-reducing zone (Froelich et al. 1979).

The near-surface oxidation of Fe(II) to Fe(III) and the subsequent formation of Fe(III) oxides, is understood to preferentially incorporate heavier isotopes into authigenic Fe(III) phases, leaving behind lighter Fe(II) (e.g., Welch et al. 2003). Accordingly, a trend towards lower porewater δ^56^Fe is observed from ~6 cm depth (~ −1.0‰) towards the sediment surface (~ −3.0‰) during both seasons, indicative of oxidative Fe(II) removal, and recycling during DIR (Severmann et al. 2006; Homoky et al. 2009). A return to higher porewater δ^56^Fe in the uppermost sediment layer was observed during late spring and similar trends have been observed in sediment cores collected from shelf-slope sediments in the South East Atlantic (Homoky et al. 2013) and in the North Sea (Henkel et al. 2016). Henkel et al. (2016) reason that oxidative precipitation of Fe preferentially removes light isotopes, as proposed by Staubwasser et al. (2013), due to environmental variances in kinetic and equilibrium isotope fractionation processes compared to experiments (e.g. Welch et al. 2003). Whereas Homoky et al. (2013) reasoned that a transition to higher δ^56^Fe towards the sediment surface resulted from mixing with an isotopically heavier and more stable Fe source, that has a relatively low dFe concentration. In both scenarios, a potential role for organic complexion of Fe exists. If such organic complexes were to stabilise a fraction of dFe across the surface oxidising zone of porewaters, the isotopic composition of the dFe pool might shift towards heavier isotopic compositions (Dideriksen et al. 2008; Morgan et al. 2010), and would resist authigenic precipitation. Accordingly, our observed trend towards higher δ^56^Fe extended from surface porewater into oxygenated core-top water and bottom water samples, while dFe concentrations steadily decreased (Fig. 3).

Porewater dFe(II) was elevated in the surface (0–1 cm) during the late spring (4.5–13.4 μM, *n* = 3) compared to late summer (0.3–1.2 μM, *n* = 3, Fig. 5; Table S3), coincident with shoaling of the OPD, linked to the deposition of organic matter during the bloom. Such seasonal variations in dFe and dMn were also reported in the water column of the North Sea and attributed to bloom-promoted release from sediments (Schoemann et al. 1998). Unexpectedly, our observed late spring surface porewater dFe(II) values are in the same range as surface sediment dFe concentrations (~7 μM) from the high-carbon accumulating and low-oxygen Oregon Shelf (120 m water depth, Homoky et al. 2009), where substantial benthic fluxes of dFe have been measured. Albeit, our reported values remain lower than surface sediments underlying near-anoxic waters (e.g. ~100 μM dFe(II), (Severmann et al. 2006, 2010). Dissolved Fe concentrations in overlying bottom waters (20 nM, with 15 nM as dFe(II); Table S4) were 1–5 orders of magnitude lower than the upper centimetre of porewater, and even lower in bottom water 10 m above the seafloor (5–10 nM; Fig. 3). The presence of Fe(II) ranged from ~70% of dFe in directly overlying bottom water at our study site (Table S4) to ~4% at 10 m above sea floor (Fig. 6; Table S5), indicating that a significant fraction of upward diffusing Fe(II) is able to escape the oxidative trap in the surface sediments and enter the water column.

### Impact of water column oxygen on release of benthic Fe(II)

Large benthic fluxes of dFe to the water column are widely reported in oxygen deficient zones and are on the order of 100–1000 μmol m^−2^ day^−2^ (Homoky et al. 2016 and references therein). These observations enable an empirical assessment of the impact of oxygen concentration on the release of Fe(II) from seafloor sediments (Dale et al. 2015). Parts of the UK shelf, other than our study site, seasonally undergo modest periods of reduced-oxygen concentration (e.g., 160–200 μmol l^−1^, compared to 280–310 μmol l^−1^ at other times of the year; Greenwood et al. 2010). To examine the likely impact of such changes in bottom water oxygen on the release of dFe from our study site, sediment cores and bottom water were sealed from the atmosphere, so that benthic respiration processes would draw down oxygen from the overlying water into the sediment. This resulted in similarly reduced dissolved oxygen concentrations at t = 0 h of ~150 and ~120 µM for late spring and late summer, respectively, but the accumulation of dFe in bottom waters was substantially different (Fig. 7). High dFe concentrations (up to 240 nM) were measured in the late spring experiment, while in the late summer dFe concentrations only reached ~25 nM. This indicates that seasonal differences in near surface pore water dFe concentration (Fig. 5) and OPD (Fig. 2) are important controls on the release of dFe to bottom waters.

During the late spring, aeration of the incubated core top water induced rapid oxidation of Fe(II) and removal of dFe from solution (Fig. 7). However, for both seasons the residual concentration of dFe is in the range 25–30 nM, of which 30–50% is present as Fe(II) despite reaching saturated oxygen concentrations—roughly 10 times greater than dFe concentrations reported for bottom waters at this site (5.4–10 nM, ~10 m above seafloor; Figs. 3, 6; Tables S4, S5). Seawater Fe(II) oxidation kinetics predict nearly all Fe(II) should be oxidised to Fe(III) in our experiments in just a few minutes (Millero et al. 1987) and for this reason it has been generally assumed that oxic shelves are not a significant source of Fe to the overlying water column. However, the observation of Fe(II) present in oxic waters over a period of >2 days suggests that rapid oxidation and fallout of Fe oxides is inhibited, due to some sort of Fe(II) and Fe(III) stabilisation. It is possible that organic carbon present during the late spring period, not only enhances the release of dFe, but also enhances the formation of organic ligands that are able to bind with Fe(II) and Fe(III) and serve to reduce the oxidative removal of dFe.

### Fe(II) oxidation kinetics in core-top and water column seawater

The oxidation kinetics of Fe(II) were investigated in water column samples and in seawater overlying sediment cores (Fig. 8). Oxidation rates in core top water were nearly twice as slow as in bottom waters, which themselves were more than 5 times slower than our empirical predictions (respective [Fe(II)] half-lives were 41, 23 and 3.8 min after Millero et al. 1987). Further, the observed Fe(II) concentrations do not rigorously follow first order kinetics. Such behaviour has been observed in hydrothermal vent plumes, suggesting some stabilisation of the reduced form of Fe, possibly through organic complexation (Statham et al. 2005). Evidence for stabilisation of dFe(II) in marine systems by organic ligands has been observed in estuarine waters (Hopwood et al. 2015), in previous shelf sediment incubation experiments (Homoky et al. 2012), and in bottom waters adjacent to the continental margin (Bundy et al. 2014). Thus, most likely, the complexation of Fe to organic ligands plays an important role in stabilising sediment-derived Fe delivered to the water column. Laboratory studies have shown that a range of simple organic molecules can impact Fe(II) oxidation rates, and whilst some had no effect, others directly or indirectly slowed the net oxidation rate (Santana-Casiano et al. 2000). It is also possible that inorganic complexes such as sulphides could stabilize Fe(II) in solution in the form of Fe sulphide nanoparticles (Yücel et al. 2011), but evidence for this in shelf systems has not been demonstrated.

The high residual dFe(II) concentrations at the end of incubation experiments show that something—most likely organic complexation—must routinely inhibit the oxidation of Fe(II) and maintain a fraction of dFe(II) in solution. Core top waters sampled throughout our study had consistently elevated dFe(II) and dFe concentrations (up to 14 and 21 nM, respectively, Table S4). The diffusion experiment in the late summer showed no significant increase or decrease in dFe(II) (0.8 ± 0.5 nM, *n* = 6) or dFe (2.8 ± 0.6 nM, *n* = 6) over a period of 6 days in core-top water (Figure S2). Therefore, elevated dFe(II) and dFe concentrations found in sampled core top waters must reflect an effectively stable form of Fe. Most importantly, dFe(II) concentrations in core top water were substantially higher than dFe concentrations in the overlying water column, indicating dFe(II) most likely originates from the sediments and provides a source of dFe to the water column even in the late summer.

Organic complexation is also able to keep Fe(III) in solution above solubility-controlled values, within the available ligand capacity. Evidence for Fe(III) ligand production in sediments has been provided for an estuarine system (Jones et al. 2011). As the Fe(II) in the proposed organic ligands is oxidised, it may remain associated with the ligand complex, converting to Fe(III)–L complexes, which may be much stronger than Fe(II)–L complexes. Alternatively, Fe(II) precipitation to Fe(III)-oxyhydroxide nanoparticles may constitute a colloidal fraction of dFe with or without organic complexes (e.g., Raiswell and Canfield 2012).

### Modelling of Fe(II) fluxes from sediments to an oxic water column

**Fig. 9:**
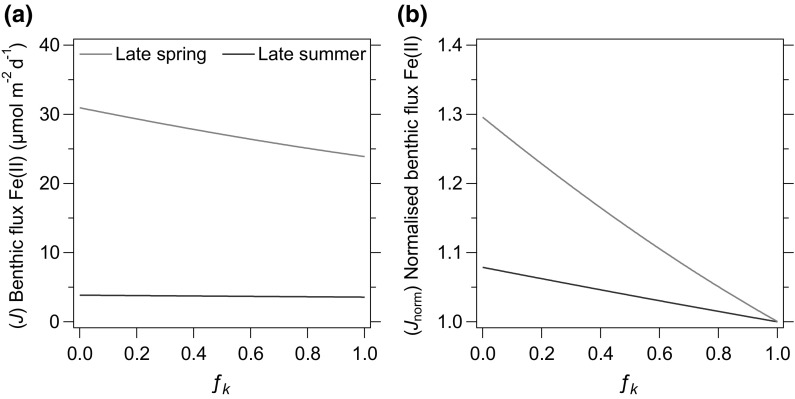
Calculated diffusive fluxes of Fe(II) to bottom water. **a** Benthic fluxes calculated as a function of fractional (*f*) strength of the Fe(II) oxidation rate constant *k*. Where *f*_*k*_ = 1, *k* follows empirical dependencies of Fe(II) oxidation rate in seawater (Millero et al. 1987). Where *f*_*k*_ is <1, Fe(II) oxidation is inhibited to simulate the presence of Fe(II)-stabilizing ligands. **b** Benthic Fe(II) flux normalized to its calculated value in the absence of any simulated ligands (*f*_*k*_ = 1). These theoretical considerations illustrate how benthic Fe(II) fluxes are greatest in late spring, and that ligands may serve to enhance the diffusive flux of dissolved Fe species released from sediments by up to 30% in late spring and 8% in late summer

Organic complexes may inhibit the oxidative precipitation of dFe(II), and could therefore increase the diffusive flux of dFe(II) across the oxic surface layer of shelf sediments to the overlying water column. We consider the impact of organic complexes using a 1-dimensional, steady-state, transport-reaction model to calculate diffusive fluxes of Fe(II) from porewater to the water column. Our approach follows Raiswell and Anderson (2005), which is used elsewhere to evaluate pore water fluxes of Fe(II) (Homoky et al. 2012, 2013; Wehrmann et al. 2014). To simulate the presence of organic ligands we simply use a fraction (*f*) between 0 and 1 of the Fe(II) oxidation rate constant (*k*), to calculate the diffusive fluxes of Fe(II) based on site A sediment characteristics (Fig. 9, see Supplementary Information).

In the absence of any Fe(II)-stabilising ligands (*f*_*k*_ = 1) a diffusive flux of 24 µmol Fe(II) m^−2^ day^−1^ is calculated from Site A under late spring conditions, where OPD was 3.3 mm, pH 7.25 and near-surface Fe(II) concentration was 6.1 μM (averaged at 0.5 cm, *n* = 3). A smaller flux of 3.6 µmol m^−2^ day^−1^ is calculated for late summer conditions (OPD = 4.1 mm, pH 6.88, Fe(II) = 0.9 μM, *n* = 3). If Fe(II) oxidation was prevented in the surface sediment (*f*_*k*_ → 0) these fluxes would increase by 30 and 8% to 31 and 3.9 µmol m^−2^ day^−1^, respectively. Although we only have ionic diffusion coefficients available for our treatment of Fe(II)–Ligand complexes, Fe(II)–stabilizing ligands at Site A have a clear potential to impact diffusive fluxes. However, by stabilising dissolved species of Fe, their impact in the overlying water-column will likely be even more significant for benthic inputs. Diffusive fluxes of Fe(II) in the late spring period would provide up to 0.3 nmol l^−1^ day^−1^ throughout an evenly-mixed 110 m water column, compared to <0.04 nmol l^−1^ day^−1^ in the late summer period. Our theoretical approach considers only diffusive transport, yet transfer of Fe(II) from pore waters could be further enhanced by advective transport due to physical mixing in the water-column, bio-turbation and bio-irrigation or anthropogenic disturbance to surface sediments.

A previous study of benthic Fe cycling in depositional areas of the North Sea found no dFe flux from sediments to overlying water using a steady state reaction-diffusion model, but found 20–210 µmol m^−2^ day^−1^ when calculating diffusive dFe fluxes from measured porewater profiles modelling simple diffusion that ignored Fe(II) oxidation (Slomp et al. 1997). We can compare our diffusive Fe(II) fluxes from porewaters with those predicted by a recent global assessment of benthic Fe flux measurements from benthic chambers. Dale et al (2015) describes the dependence of benthic Fe flux on the rate of organic carbon oxidation in sediments and bottom water oxygen concentrations based on a compilation and regression of all known determinations. Where our organic C oxidation rates are calculated to be 8.6–11.9 mmol m^−2^ day^−1^ and bottom water oxygen is 267–252 µM, benthic Fe fluxes for our study site are estimated to be 8.0 µmol m^−2^ day^−1^ in late spring and 5.5 µmol m^−2^ day^−1^ in late summer—slightly higher than our late summer determination of 3.6–3.9 µmol m^−2^ day^−1^, but less than our late spring determination of 24–31 µmol m^−2^ day^−1^. To a first approximation, this is a favourable comparison, and it is not unreasonable that individual study sites will have benthic Fe fluxes that deviate from the averaged relationships described by Dale et al. (2015). However, it is also clear that well-oxygenated ocean margins have been largely absent from the compiled benthic Fe flux data used to parameterise ocean biogeochemical models to date (Homoky et al. 2016), hence there is potential for an underestimated contribution of dFe from oxic ocean margins.

### Implications of benthic Fe(II) fluxes to an oxic water column

The dFe(II) stabilisation outlined above may enhance and maintain dFe(II) fluxes to the overlying water column. A study of the shelf and slope in the Bay of Biscay, south-west of the coast of Brittany found that labile dFe(II) species account for >8% of dFe species in bottom waters of the shelf break, and suggested that benthic processes (resuspension and diagenesis) represent important sources of dFe(II) and dFe, increasing the availability of Fe to microorganisms (Ussher et al. 2007). Elevated dFe(II) near the sea floor at Site A was also observed in July 2015 (Fig. 6) and represents ~4% of the dFe pool (Birchill, personal communication). The steep increase in dFe(II) concentrations towards the seafloor is consistent with our evaluation of a sedimentary source, although release of dFe(II) from the degradation of organic matter in the water column may also contribute to bottom water dFe(II) maxima.

**Fig. 10:**
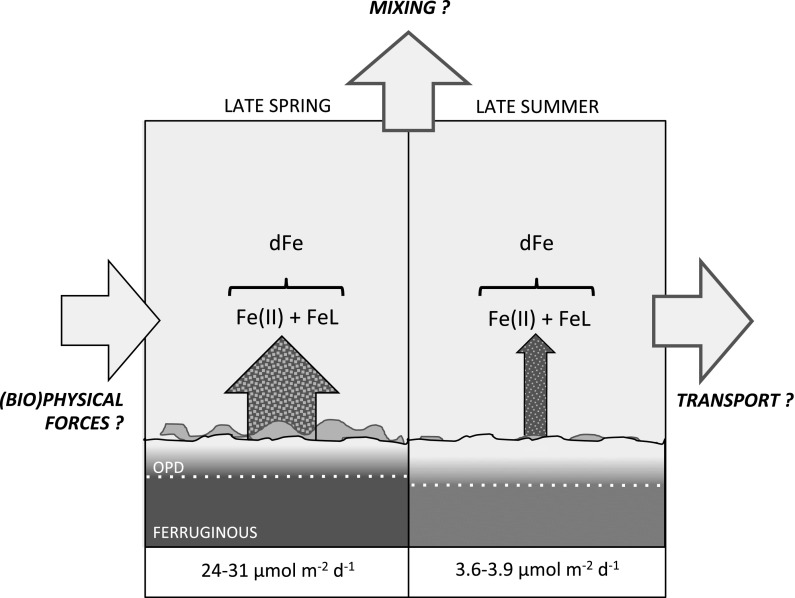
Seasonal diffusive Fe(II) fluxes from the Celtic Sea Shelf and the proposed influence of stabilizing ligands. Calculated fluxes of Fe(II) to *bottom water* are indicated by *arrow widths*, and the potential influence of Fe(II)-stabilizing ligands by the density of *green shading* within *arrows*. Diffusive fluxes of Fe(II) which ignore the influence of ligands are smaller and correspond to minimum flux values described for each season. A shoaling of the oxygen penetration depth (OPD), increase in the surface porewater inventory of dFe(II) and an increase in the diffusive flux of dFe(II) to bottom waters coincides with the enhanced supply and decomposition of phytodetritus at the seafloor in late spring. An apparent 10–20 fold reduction in Fe(II) oxidation rates over empirical predictions observed in our study was attributed to the stabilizing influence of Fe(II)-complexing organic ligands at the seafloor. The extent to which ligands may moderate benthic Fe fluxes will further depend on their size, abundance, stability and diffusive properties, which were not determined in this study. Dissolved Fe delivered to bottom water will undergo continued oxidation of Fe(II) to Fe(III) in addition to particle-adsorption and aggregation processes. The true magnitude of dissolved Fe species released to bottom waters, will further reflect the influence of biological and physical disturbances to the sediment-water interface and transport mechanisms within the benthic boundary layer, which may further enhance exchange rates, but are neglected in our assessment

Whilst there appears to be a low background diffusive input of Fe from the sediments throughout the year (Fig. 9; Figure S2), the deposition of C in biogenic debris from the upper water column after the spring bloom is a major driver for additional inputs of Fe to porewaters, as was previously hypothesised (Schoemann et al. 1998). The degradation and turn-over of this organic material at the seafloor appears to happen within a 2-month period. Following release of Fe in the late spring, sediments gradually reset to pre-bloom conditions. This view of the carbon flux to sediments being a major driver for iron release supports predictions from Elrod et al. (2004) and the revaluations by Dale et al. (2015). It is shown that the Fe flux is dependent on the position of the redoxcline within the sediment and the availability of organic material at the seafloor, with the stabilisation of dFe(II) by ligands acting as a further mechanism to enhance dFe transfer to the water column (Fig. 10). The findings discussed here apply to cohesive shelf sediments. However, sandy mud only covers 0.8% of the seafloor in the Celtic Sea. Sandy sediments, on the other hand, cover a large fraction of the seabed (16.5%, Thompson et al. submitted) and are also affected by seasonal inputs of organic matter, where organic Fe complexation could mediate benthic exchanges of Fe. This unconsolidated coarse sediment contains less organic carbon, but is much more permeable and so may host important advection-dominated exchange of dFe in shelf settings.

Based on our findings, we suggest that temperate shelf seas equivalent to the Celtic Sea need to be more explicitly represented in future ocean biogeochemical models, where ligand-mediated benthic exchanges of Fe occur in response to seasonal phytoplankton blooms. In such environments, Fe flux predictions based on previous benthic chamber studies (e.g., Dale et al. 2015) might underestimate the true magnitude of dissolved Fe input to the shelf seas. Although many sediment types across the Celtic Sea receive seasonal inputs of organic matter, benthic Fe fluxes in cohesive and non-cohesive sediments will be controlled by distinct diffusion-advection regimes for porewater solutes. Animal activity, waves, tides and human-induced disturbances of shelf sediments will all impact transport processes, but they are also ill quantified. An appraisal of exchange processes and rates across coarser and more permeable sediments will support a more rigorous scaling-up of our findings across the Celtic Sea, to quantify the impact of ligand-mediated benthic fluxes to oxic shelf seas. Such a result, once seasonal perturbations to benthic oxygen and to carbon dynamics and ligand-sustained fluxes of Fe are properly accounted for, is likely to reveal that a larger amount of Fe is released from oxic shelf sediments, which are estimated to typify most of the ocean-continent boundary, especially in large areas of the Atlantic, Arctic and Southern Oceans (Homoky et al. 2016), than previously assumed.

## Conclusions

Porewater dFe in cohesive sediments underlying an oxic shelf is mainly present as Fe(II) in the soluble size fraction. This implies that porewater Fe(II) is in the ionic form or complexed to ligands in the soluble size fraction. Porewater Fe(II) is produced at ~6 cm below the surface via DIR, and is partially trapped in surface sediments as insoluble Fe(III) oxide phases during upward diffusion, leading to low concentrations of residual dFe(II) in surface porewaters with a characteristically light isotopic composition (δ^56^Fe down to −3‰).

Even though large amounts of porewater dFe are lost to oxidation, the studies described here provide evidence for a significant release of dFe as Fe(II) from cohesive sediments underlying oxic waters on shelves in temperate systems. Stabilisation of dFe(II) in surface sediment porewaters appears to be an important factor for diffusion across the surface oxidizing layer of sediments, and most especially for the fate of dFe(II) species that are subsequently entrained in the water-column and contribute to elevated dFe concentrations on this oxic shelf. The deposition of phytoplankton debris at the seafloor provides a boost to the release of Fe through the decomposition and remineralisation of organic matter, as well as increased diagenetic release from sediments under changing redox conditions.

The work reported here provides a mechanistic explanation for the elevated dissolved water column Fe found overlying oxic shelves, relative to the open ocean. Given the global extent of oxic shelves, our work implies a larger shelf source of dFe to the ocean might exist than is predicted from existing compilations of benthic Fe fluxes that do not resolve the influence of organic complexation of Fe originating from shelf settings. Detailed studies of organic stabilisation processes in these and other ocean-sediment settings are required to improve our knowledge of the role and sensitivity of sedimentary sources of Fe linked to marine biogeochemical cycles.

## Electronic supplementary material

Below is the link to the electronic supplementary material.

Supplementary material 1 (DOCX 363 kb)

Supplementary material 2 (XLSX 14 kb)

Supplementary material 3 (XLSX 17 kb)

Supplementary material 4 (XLSX 12 kb)

Supplementary material 5 (XLSX 10 kb)
