# Supplementary material for: Stability of dissolved and soluble Fe(II) in shelf sediment pore waters and release to an oxic water column
Source: Biogeochemistry. 2017 Feb 27;135(1):49–67. doi: 10.1007/s10533-017-0309-x (PMC6961528; doi:10.1007/s10533-017-0309-x)
Supplement: Supplementary file 1 — Supplementary material 1 (DOCX 363 kb) [file 10533_2017_309_MOESM1_ESM.docx]

Supplementary Information

Stability of dissolved and soluble Fe(II) in shelf sediment pore waters and release to an oxic water column

Klar, J.K.^a,b*^, Homoky, W.B.^c^, Statham, P.J.^a^, Birchill, A.J.^d^, Harris, E.L.^a^, Woodward, E.M.S.^e^, Silburn, B.^f^, Cooper, M.^a^, James, R.H.^a^, Connelly, D.P.^g^, Chever, F^a^, Lichtschlag, A.^g^, Graves, C.^a^

^a^ Ocean and Earth Science, University of Southampton, National Oceanography Centre, Southampton, SO14 3ZH, United Kingdom.

^b^ Present address: LEGOS, Université de Toulouse, CNES, CNRS, IRD, UPS, 14 Avenue Edouard Belin, 31400 Toulouse, France.

^c^ University of Oxford, Department of Earth Sciences, South Parks Road, Oxford, OX1 3AN, United Kingdom.

^d^ School of Geography, Earth and Environmental Science, University of Plymouth, Drake Circus, Plymouth PL4 8AA, United Kingdom.

^e^ Plymouth Marine Laboratory, Prospect Place, The Hoe, Plymouth, PL1 3DH, United Kingdom.

^f^ Centre for Environment, Fisheries and Aquaculture Science, Pakefield Road, Lowestoft, NR33 0HT, United Kingdom.

^g^ National Oceanography Centre, University of Southampton Waterfront Campus, European Way, Southampton, SO14 3ZH, United Kingdom.

^*^Corresponding author, email [jessica.klar@legos.obs-mip](mailto:jessica.klar@legos.obs-mip)

# Analytical methods

## Oxygen depth-profiles and time-series

A suite of Unisense O_2_ micro sensors was used to measure the concentration of dissolved O_2_ at 100-200 μm depth-intervals, and to monitor changes over time during Fe(II) oxidation experiments. Assessments of O_2_ penetration depth (OPD, limit of O_2_ detection ≤ 0.3 μM) were based on 2-4 profiles from each sediment core of the 3 collected in each season (Homoky et al. 2011; Homoky et al. 2013). Briefly, microsensors were calibrated using two end-point O_2_ saturation values: 100 % (air saturated) and 0 % (N_2_-flushed/O_2_-purged or dithionite) seawater solutions were matched in temperature and salinity to bottom waters. Oxygen saturation was converted to molarity following its empirical dependence on salinity and temperature using SensorTracePro software.

## Ship-board Fe(II) and total Fe analyses

The concentrations of Fe species - Fe(II) and Fe(II) plus Fe(III) (hereafter total Fe) – were determined in the dissolved and soluble size fractions of porewater samples using the Fe(II)-complexing ferrozine ligand (Sigma-Aldrich). The absorbance of the Fe(II)-ferrozine complex was measured spectrophotometrically at 562 nm before and after reduction of Fe(III) to Fe(II) by ascorbic acid (Sigma-Aldrich, TraceSELECT®) to determine respective Fe(II) and total Fe concentrations (Stookey 1970; Viollier et al. 2000). For sub-surface porewaters, where Fe concentrations were expected to be >1 μM, a 0.5 ml subsample was pipetted into 1.5 ml of 2 mM Ferrozine for Fe(II), and into 1.5 ml of 2 mM Ferrozine plus 0.2 ml of 10 mM ascorbic acid for total Fe. For surface porewaters and incubation experiment samples, where Fe concentrations were expected to be < 1 μM, a 1 ml subsample was pipetted into 100 μL of 5 mM Ferrozine in 1 M ammonium acetate buffer for Fe(II), and into an equal amount of Ferrozine-buffer plus 100 μL 10 mM ascorbic acid for total Fe. Concentrations > 1 μM were analysed in a 1 cm quartz cell on a spectrophotometer (ATI Unicam 8625). Concentrations < 1 μM were measured on a 250 cm 3000 Series Liquid Waveguide Capillary Cell (LWCC, World Precision Instruments) coupled to a tungsten LS-1 light source (Ocean Optics) and a USB4000 fibre optic spectrometer (Ocean Optics) (Waterbury et al. 1997). Fe(II) standards were prepared by diluting ammonium iron(II) sulfate hexahydrate (Sigma-Aldrich, purum p.a. grade) to match the concentrations of chemicals in the samples. On the UV-vis spectrophotometer, the limit of detection (LOD, three times the standard deviation of the blank) was 0.3 μM Fe(II) and the blank was 0.25 μM Fe(II). The typical relative standard deviation (obtained by measuring replicates) was 2 % for > 10 μM and up to 5 % below 4 μM. For the LWCC, the LOD was 0.7 nM, the blank was 6 ± 4 nM and the typical relative standard deviation was < 5 %.

## Analyses of labile Fe(II) in seawater samples

During cruise DY033 (11^th^ July – 8^th^ August 2015) water column samples were collected with a Ti-CTD. Cast subsamples for labile Fe(II) were transferred into acid-washed and pre-rinsed LDPE vials. The samples were filtered inline by peristaltic pumping directly through a syringe filter (0.2 μm-pore size, PES membrane, Nalgene), that had been flushed (1 hour) with filtered seawater, into a flow injection chemiluminescence (FI-CL) analyzer. Samples were stored at 4 ^o^C in the dark and analysis took place within 20 minutes of sampling. Labile Fe(II) was determined by inline solid phase preconcentration onto an 8-hydroxyquinoline resin at pH 5.2, followed by acid elution and luminol chemiluminescence detection (Bowie et al. 2002; Ussher et al. 2007). The analyser was preconditioned by multiple analyses of a ‘control’ seawater sample and blank injections.

Calibration was performed by standard additions of Fe(II) to pH adjusted filtered surface seawater (pH 5.2). The limit of detection of this method (defined as three times the standard deviation of the blank) was 15 pM Fe(II) and the blank was 25 pM Fe(II). The average relative standard deviation, obtained from triplicate analyses, for all samples above the LOD was 6.7 %. The measurement was termed ‘‘labile Fe(II)’’ because the exact speciation of the iron complexes is unknown, and the term Fe(II) may imply that only inorganic Fe(II) species were determined.

## Fe isotope analyses

For isotopic measurements of Fe in seawater samples and core-top waters a two-step procedure was used (Lacan et al. 2010; Conway et al. 2013). For a target quantity of 200 ng Fe, samples were pre-concentrated using NTA Superflow resin (Quiagen), and then purified by anion exchange chromatography (using AGMP-1 resin, BioRad). Porewater samples contained sufficient Fe such that only the purification step was needed. Samples were then analysed on a Neptune *Plus* multi-collector inductively coupled plasma mass spectrometer (MC-ICP-MS) (Thermo Scientific) in high resolution mode. Mass fractionation was corrected by adding a ^57^Fe/^58^Fe double spike to samples prior to chemical processing. Isotope ratios are expressed in delta notation relative to the average value of the reference material IRMM-014 (Institute for Reference Materials and Measurements) determined during the same analytical session: δ^56^Fe (‰) (= [(^56^Fe/^54^Fe)_sample_/(^56^Fe/^54^Fe)_IRMM-14_ – 1] × 1000). The procedural blank, determined on an Element 2XR ICP-MS (Thermo Scientific) was 4.3 ng Fe for the preconcentration step and 0.5 ng Fe for the purification step. The blank contributed < 4 % Fe to the sample. The Fe isotopic haematite standard ETH (Eidgenössische Technische Hochschule, Zürich) was analysed regularly during analytical sessions and was 0.52 ± 0.04 ‰ (2 SD, *n* = 38), compared to a consensus value of 0.52 ± 0.08 ‰ (2SD, *n* = 80; Lacan et al., 2010). The analytical procedure was validated by taking aliquots of ETH reference material through the purification procedure (0.55 ± 0.05 ‰, 2 SD, *n* = 2).

## Analyses of Mn concentrations in porewaters

Porewater Mn concentrations in soluble and dissolved size fractions were determined in diluted samples (20 to 100 fold in 0.3 M thermally distilled HNO_3_) on a quadrupole ICP-MS (X-Series, Thermo Scientific). The blank was < 0.8 nM, the LOD was 0.2 nM and the typical relative standard deviation was 2 %.

## Leachable Fe and Mn phases in sediments

In order to examine phase associations of Fe and Mn in solid sediment phases, two leaching schemes were applied to one core per season. An ascorbic acid leach (Raiswell et al. 2010) was applied, which extracts the easily reducible oxide phase such as amorphous ferrihydrite but not the more crystalline oxide phases. Additionally, an acetic acid-hydroxylamine-HCl (H-HCl) leach (Berger et al. 2008) was also used to extract other amorphous oxide phases as well as ferrihydrite. For detailed method descriptions, the reader is referred to the references cited above. Fe and Mn in the leach solutions were determined using an inductively coupled plasma optical emission spectrometer (ICP-OES, iCAP6000 Series, Thermo Scientific). For Fe, the blank was < 0.06 μg/g, the LOD was < 4 μg/g and the typical standard deviation was ~20 μg/g. For Mn, the blank was < 0.02 μg/g, the LOD was < 0.6 μg/g and the typical standard deviation was 4 μg/g.

## Nutrient and sulphide analyses

Nutrient concentrations in water column samples, in sediment porewaters and experiments, were all analysed on board using a Bran and Luebbe segmented flow colorimetric auto-analyser (Woodward and Rees 2001). Sampling and handing techniques were carried out according the International GO-SHIP nutrient manual recommendations (Hydes et al. 2010). Nutrient reference materials (KANSO Japan) were run each day for quality control. The typical relative standard deviation was 2-3 %; and the limits of detection were 0.02, 0.01, and 0.05 µmoles L^-1^ for “nitrate plus nitrite”, nitrite, and ammonia, respectively. Nitrate was obtained by subtracting nitrite from the “nitrate plus nitrite” measurement. Sulphide was measured using the colorimetric technique of Cline (1969), and the detection limit was 1 µM.

## POC & PON in sediments

Particulate organic carbon (POC) and nitrogen (PON) were determined using a Carlo-Erba CHNOS analyser. The procedure followed Nieuwenhuize et al. (1994), in which the carbonate in samples contained in silver cups is removed by acidification with HCl before measuring POC, and inorganic C is given by the difference between a total C value and the POC measured. Precision was 6.6 % RSD at 1.5 % POC and 2.4 % RSD at 0.13 % PON. Estimated detection limit for C was 3.6 µg and all sample carbon contents were well above this value (Figure S1). Elemental Microanalysis Soil was used as a standard and monitored regularly.

(b)

(a)

Figure S1: Distributions of total nitrogen (TN), organic carbon (OC) and carbonate during late spring conditions (a); and a seasonal comparison of the distribution of organic carbon (b) in sediments at Site A.

## pH measurements in experiments

All pH electrodes were calibrated daily using a Certified Reference Material (TRIS pH buffer in synthetic seawater) supplied by Andrew Dickson, University of California (Nemzer and Dickson 2005; Silburn et al. submitted).

# Benthic Fe diffusion experiment

Three replicate cores were collected from site I (muddy sand, ~ 50° 34.5557 N, 7° 6.3161 W, Thompson et al., submitted). Sediments at this site are characterized by a higher sand content, compared to Site A. A Milli-Q water rinsed control core tube containing no sediment was set up as a control. Original core top water was subsampled before carefully being syphoned off. A known amount (1 L) of clean unfiltered seawater (collected with the Ti-CTD at Site I) was slowly added to each of the 4 cores. This water had ~ 1 nM dFe and dFe(II) below LOD (< 0.7 nM). Core top water was kept oxygenated and in the dark at all times and was subsampled at several time intervals for dFe, dFe(II) and pH using Teflon tubing and a syringe. The experiment was carried out in a temperature-controlled lab at 11.5 to 11.7 °C.

The control core had dFe and dFe(II) concentrations below LOD, most likely due to adsorption onto the walls. Original core top water contained 10 ± 9 nM dFe and 8 ± 9 nM dFe(II) (Figure S2). After the replacement with clean water, dFe and dFe(II) concentrations were significantly lower and did not change significantly over time (average over 6 time points was 2.8 ± 0.6 nM dFe and 0.8 ± 0.5 nM dFe(II); Figure S2). The average water pH was 7.98 ± 0.01 (1SD, *n* = 4).

Figure S2: Concentrations of dFe(II) and total dFe in core top water, to mimic the diffusion across the sediment-water interface, carried out at Site I (muddy sand, Thompson et al., submitted) in late summer conditions. Original Core top water (represented by black and empty triangles for dFe and dFe(II), respectively) was exchanged with bottom water at time 0. Error bars represent the standard deviation of three replicate measurements in three replicate experiments.

# Calculation of benthic Fe(II) flux from porewaters

We follow Raiswell and Anderson (2005) who describe the flux of Fe(II) to bottom water as a function of vertical diffusion and oxidative-loss in surface sediments beneath an oxygenated water column. This approach has previously been used to calculate the steady-state diffusive flux of dissolved Fe to oxygenated waters from subtropical, temperate and high-latitude ocean margin sediment pore waters (e.g., Homoky et al. 2012; Homoky et al. 2013; Wehrmann et al. 2014). For a consideration of the parameterisation of Fe(II) oxidation rate in these calculations (e.g. due to NO_3_^-^, pH) and an inter-comparison of the approach to Fe flux determinations by *in situ* benthic chambers, see Homoky et al. (2012). Equation S1 describes the vertical diffusive flux *(*$J$) of Fe(II) from sediment pore water to bottom waters in units of mol cm^-2^ s^-1^:
 $J = \frac{\varphi(D_{s}k_{1})0.5C_{p}}{sinh[(k_{1}/D_{s})0.5L]}$ , (Eq. S1)

where $\varphi$ is sediment porosity (with assumed uniformity), $L$ is the thickness (cm) of the oxygenated surface layer (equal to oxygen penetration depth plus diffusive boundary layer thickness), and $C_{p}$ is the concentration (g cm^-3^) of Fe(II) in the pore water beneath $L$.

The rate constant for Fe(II) oxidation is represented by $k_{1}$ (s^-1^, Equation S2):

$k_{1} = k[O_{2}]{[{OH}^{-}]}^{2},$ (Eq. S2)

and is a function of bottom water O_2_ concentration (mol L^-1^), pH, and a value $k$ (s^-1^) derived from the temperature, $T$, (°K) and salinity ($I$ = ionic strength) dependence of Fe(II) oxidation rate in seawater (Equation S3) after Millero et al. (1987):

$log k = 21.56-\frac{1545}{T}-3.29I^{0.5}+1.52I$, (Eq. S3)

We also derive a diffusion coefficient of pore water Fe(II) in muddy shelf sediments after Raiswell and Anderson (2005) in Equation S4, where $D_{s}$ (cm^2^ s^-1^) is a function of $\varphi$, corrected for tortuosity, and temperature, $T$ (°C):

$D_{s} = \varphi1.7(3.31 + 0.15T){10}^{-6}$. (Eq. S4)

Simulated impact of Fe(II)-stabilising ligands on benthic Fe(II) flux

We consider the impact of Fe(II) stabilised by ligands (Fe(II)-L) present in pore water, on the steady-state diffusive fluxes of Fe from shelf sediments. In our assessment we must assume Fe(II)-L has a diffusion coefficient equal to ionic forms of Fe(II) in solution. Such a simplification of reality likely overestimates the true coefficient of Fe(II)-L diffusion. However, even though such properties are not truly known, comparatively large complexes of some other trace elements (e.g. Cu and Pb) are estimated to diffuse just ~25 % slower than free ions in solution (Phillips and Ellis 1970; Scally et al. 2006). In the absence of known values, and given that we observe dFe(II) almost entirely in a soluble size fraction, we consider an application of ionic diffusion coefficients in this context to be reasonable.

We simulate the presence of Fe(II)-L by inhibiting Fe(II) oxidation rates. To do so, we use a variable fraction ($f$) of $k_{1}$ from 1 towards 0, so Equation S1 becomes S5:

$J = \frac{\varphi(D_{s}{fk}_{1})0.5C_{p}}{sinh[(fk_{1}/D_{s})0.5L]}$ . (Eq. S5)

When $f$ = 1, calculation of $J$ using Equation S5 is identical to that made by S1. When $f$= <1, $k_{1}$ is reduced, inhibiting the rate of Fe(II) oxidation and precipitation from the pore water due to our simulated presence of ligands. When $f\to0$, Fe(II) is effectively stable in the pore water for the maximum diffusive length scale we have assessed (0.47 cm/1.8 days). Calculated fluxes ($J)$ are presented in Supplementary Table S1, where $f=1$ and $f\to0$. Refer to main article and Figure 9 for further details.

# Data repository (see accompanying data files)

Table S2: Concentrations of Fe and Mn in leachable sediment phases (displayed in Figure 3), and total carbon (total C), particulate organic carbon (POC) and particulate organic nitrogen (PON) contents (displayed in Figure S1) in shelf sediments in the Celtic Sea at Site A during late spring and late summer.

Table S3: Parameters in porewaters sampled from shelf sediments in the Celtic Sea at Site A during late spring and late summer, presented in Figure 3.

Table S4: Parameters in Core Top Water (bottom water collected on top of sediment cores) and bottom water collected with the titanium rosette in the Celtic Sea at Site A during late spring and late summer, presented in Figure 3.

Table S5: Water column dFe(II), sFe and dFe at site A in the Celtic Sea in July 2015 (Cruise DY033), presented in Figure 6. Water depth was 106 m.

# References

Birchill AJ, Lohan MC, Milne A, Ussher SJ, Worsfold PJ, Hopkins J, Sharples J et al. (in prep) Iron deplete waters in the Celtic Sea following seasonal stratification. Geophysical Research Letters

Bowie AR, Achterberg EP, Sedwick PN, Ussher S, Worsfold PJ (2002) Real-Time Monitoring of Picomolar Concentrations of Iron(II) in Marine Waters Using Automated Flow Injection-Chemiluminescence Instrumentation. Environmental Science & Technology 36:4600-4607 doi:10.1021/es020045v

Conway TM, Rosenberg AD, Adkins JF, John SG (2013) A new method for precise determination of iron, zinc and cadmium stable isotope ratios in seawater by double-spike mass spectrometry. Anal Chim Acta 793:44-52 doi:http://dx.doi.org/10.1016/j.aca.2013.07.025

Homoky WB, John SG, Conway TM, Mills RA (2013) Distinct iron isotopic signatures and supply from marine sediment dissolution. Nat Commun 4 doi:10.1038/ncomms3143

Homoky WB, Severmann S, McManus J, Berelson WM, Riedel TE, Statham PJ, Mills RA (2012) Dissolved oxygen and suspended particles regulate the benthic flux of iron from continental margins. Mar Chem 134:59-70 doi:10.1016/j.marchem.2012.03.003

Hydes D, Aoyama M, Aminot A, Bakker K, Becker S, Coverly S, Daniel A et al. (2010) Determination of dissolved nutrients (N, P, Si) in seawater with high precision and inter-comparability using gas-segmented continuous flow analysers. In: The GO-SHIP Repeat Hydrography Manual : A Collection of Expert Reports and guidelines. IOCCP Report No 14, ICPO Publication Series No. 134, version 1, 2010. UNESCO/IOC,

Lacan F, Radic A, Labatut M, Jeandel C, Poitrasson F, Sarthou G, Pradoux C et al. (2010) High-Precision Determination of the Isotopic Composition of Dissolved Iron in Iron Depleted Seawater by Double Spike Multicollector-ICPMS. Anal Chem 82:7103-7111 doi:10.1021/ac1002504

Millero FJ, Sotolongo S, Izaguirre M (1987) The oxidation-kinetics of Fe(II) in seawater. Geochim Cosmochim Acta 51:793-801

Nemzer BV, Dickson AG (2005) The stability and reproducibility of Tris buffers in synthetic seawater. Mar Chem 96:237-242 doi:http://dx.doi.org/10.1016/j.marchem.2005.01.004

Nieuwenhuize J, Maas YEM, Middelburg JJ (1994) Rapid analysis of organic carbon and nitrogen in particulate materials. Mar Chem 45:217-224 doi:http://dx.doi.org/10.1016/0304-4203(94)90005-1

Phillips RE, Ellis JH (1970) A rapid method of measurement of diffusion coefficients in aqueous solutions. Soil Science 110:421–425

Raiswell R, Anderson TF (2005) Reactive iron enrichment in sediments deposited beneath euxinic bottom waters: constraints on supply by shelf recycling. In: McDonald I, Boyce AJ, Butler IB, Herrington RJ, Polya DA (eds) Mineral Deposits and Earth Evolution, vol 248. Geological Society Special Publication. pp 179-194. doi:10.1144/gsl.sp.2005.248.01.10

Scally S, Davison W, Zhang H (2006) Diffusion coefficients of metals and metal complexes in hydrogels used in diffusive gradients in thin films. Anal Chim Acta 558:222-229 doi:10.1016/j.aca.2005.11.020

Silburn B, Kröger S, Parker R, Sivyer D, Hicks N, Powell C, Johnson M et al. (submitted) Benthic pH gradients in a range of shelf sea sediments linked to sediments characteristics and seasonal variability. Biogeochemistry

Stookey LL (1970) Ferrozine - a new spectrophotometric reagent for iron. Anal Chem 42:779-& doi:10.1021/ac60289a016

Ussher SJ, Worsfold PJ, Achterberg EP, Laës A, Blain S, Laan P, de Baar HJW (2007) Distribution and redox speciation of dissolved iron on the European continental margin. Limnol Oceanogr 52:2530-2539

Waterbury RD, Yao W, Byrne RH (1997) Long pathlength absorbance spectroscopy: trace analysis of Fe(II) using a 4.5 m liquid core waveguide. Anal Chim Acta 357:99-102 doi:http://dx.doi.org/10.1016/S0003-2670(97)00530-8

Viollier E, Inglett PW, Hunter K, Roychoudhury AN, Van Capellen P (2000) The ferrozine method revisited: Fe(II)/Fe(III) determination in natural waters. Applied Geochemistry 15:785-790

Wehrmann LM, Formolo MJ, Owens JD, Raiswell R, Ferdelman TG, Riedinger N, Lyons TW (2014) Iron and manganese speciation and cycling in glacially influenced high-latitude fjord sediments (West Spitsbergen, Svalbard): Evidence for a benthic recycling-transport mechanism. Geochim Cosmochim Acta 141:628-655 doi:10.1016/j.gca.2014.06.007
